# Core Promoters of Pig SOD2 Gene and Its Expression Regulation by DNA Methylation

**DOI:** 10.3390/vetsci12121133

**Published:** 2025-11-28

**Authors:** Minjun Zhao, Zhaoqi Pang, Chunhui Jia, Shunran Zhao, Wei Xia, Junjie Li, Chenyu Tao

**Affiliations:** College of Animal Science and Technology, Hebei Agricultural University, Baoding 071000, China

**Keywords:** DNA methylation, promoter, SOD2, pigs

## Abstract

In pig production, oxidative stress in the placentas of pregnant sows is one of the most important factors affecting reproductive performance. Superoxide dismutase *SOD2* is a critical component of the antioxidant system and determines the antioxidant capacity of cells. The expression of the *SOD2* gene is reportedly regulated by DNA methylation. At present, there are abundant reports on the function and structure of *SOD2*, but how DNA methylation affects the expression of *SOD2* in pigs is still unclear. In this study, we identified the core promoters of *SOD2* gene and verified the important transcription factor binding sites. Treatment of porcine placental trophoblast cells with a DNA methyltransferase (DNMT) inhibitor reduced promoter methylation and increased *SOD2* expression. Treatment of *SOD2* promoter fragments with CpG methyltransferase M.SssI reduced promoter activity. In summary, the *SOD2* core promoter is located at −275/−66 bp. Hypomethylation of the core promoter promotes the expression of *SOD2*, while hypermethylation reduces promoter activity. This study provides a theoretical basis for further investigation into the regulation of porcine *SOD2* gene by DNA methylation.

## Simple Summary

In the modern livestock production system, maintaining efficient reproductive of sows is of great significance for achieving production economic benefits and production goals. The intensification of oxidative stress may lead to a decline in the health of sows, impaired placental function, decreased birth quality of piglets, and reduced reproductive performance of sows. It is one of the main problems faced by the production industry. DNA methylation modification is involved in regulating important biological processes such as gene expression, genomic imprinting, X chromosome inactivation, cancer occurrence, embryonic development and tissue differentiation. Under certain conditions, DNA methylation can affect the expression of antioxidant enzymes in cells and regulate the cells’ response to oxidative stress. Our research group’s previous study found that in the placental tissues of sows fed with antioxidants, the expression of DNMT1 was significantly decreased, while the expression of *SOD2* was significantly increased. SOD2 plays a significant role in the antioxidant process of animals and is of great significance for improving livestock and poultry breeds and enhancing reproductive efficiency.

## 1. Introduction

In livestock production, maintaining sow performance is crucial for achieving production goals. The number of weaned piglets per sow per year (PSY) is a key indicator of pig farm efficiency and sow reproductive performance [1]. In late pregnancy and lactation, the metabolic intensity of sows increases accordingly, and the level of reactive oxygen species (ROS) in the body rises, especially in primiparous sows, whose metabolism is particularly strong. Additionally, the antioxidant capacity of sows decreases in late pregnancy, resulting in reduced plasma antioxidant enzyme activity and total antioxidant capacity [2] during the perinatal period. Oxidative stress may impair reproductive performance, leading to poor sow health, reduced litter outcomes, and decreased lactation [3]. The mammalian placenta acts as a bridge between the mother and fetus, transporting nutrients, excreting waste, and secreting steroid hormones to provide immune support for the fetus. It is one of the important organs during pregnancy [3]. Due to its high metabolic activity and frequent cell division, the placenta is highly sensitive to oxidative stress [4]. The placenta’s ability to transport nutrients from mother to fetus depends primarily on the thickness and area of the trophoblast layer, as well as the number and activity of nutrient transport carriers. The syncytiotrophoblast, located on the surface of placental villi, is particularly susceptible to oxidative stress [5]. Trophoblast cells, the main functional cells in the placenta, play a vital role in nutrient transport. Therefore, trophoblast cells from the pig placenta were selected for this study.

As the first line of defense in the biological antioxidant system, SOD is the most important member of the antioxidant enzyme system. It specifically scavenges superoxide anion radicals generated during oxidative metabolism. SOD is composed of proteins and metal ions and is classified into three main types based on metal prosthetic groups: CuZnSOD (*SOD1*), found in eukaryotes; MnSOD (*SOD2*), present in eukaryotic mitochondria and prokaryotes; and FeSOD, located in chloroplasts and prokaryotes [6,7]. The relationship between DNA methylation and antioxidant processes is a significant area of research. DNA methylation patterns are influenced by genetics, environment, diet, and disease. Oxidative stress can cause DNA damage, while DNA methylation can influence antioxidant levels [8]. For example, studies show that a high-fat diet can downregulate antioxidant enzymes like glutathione peroxidase (GPX) and SOD in the liver of mice, along with hypermethylation of their promoter regions [9]. In addition, during human aging, the expression of antioxidant enzymes like *SOD2* and catalase in white blood cells decreases, correlating with increased methylation of their promoter regions [10]. Promoter methylation can be considered an important regulatory mechanism of *SOD1* gene expression. Methylation plays a crucial role in various genes, but its presence in porcine placental trophoblast cells has rarely been reported.

This study investigated the effect of methylation on the expression of the *SOD2* gene in porcine trophectoderm cells (PTCs). Luciferase reporter assays were performed to identify the core promoter regions and examine CpG site methylation. The correlation between CpG methylation status and the expression levels of *SOD2* was clarified.

## 2. Materials and Methods

### 2.1. Culture of PTCs

The PTCs [11] used in this experiment were provided by College of South China Agricultural University. The porcine PTCs were taken out of liquid nitrogen, quickly thawed in a 37 °C water bath, resuspended in PBS, and washed twice. The supernatant was discarded after centrifugation at 1000 r/min for 5 min, and DMEM/F12 complete culture medium (containing 10% fetal bovine serum (FBS), 1% ITS, and 1% penicillin-streptomycin double antibody) was added. After resuspension, it was inoculated into a 25 cm^2^ cell culture flask (Corning) and placed in a cell incubator (37 °C, saturated humidity, 5% CO_2_) for aseptic culture. The complete culture medium was replaced every 24 h. After the fusion degree of PTCs was more than 70%, the cells were washed twice with PBS and digested with trypsin (Try-EDTA, 0.25%) at 37 °C for about 2 min. When the cells began to fall from the wall of the culture flask and float in the digestive solution, digestion was terminated with DMEM/F12 complete medium containing serum, and the cells were centrifuged at 1000 r/min for 5 min. The supernatant was re-suspended in complete medium and then transferred into a new 7.5 cm^2^ culture flask for passage. The PTCs were passaged more than 3 times, and the cell morphology was normal and stable. When the number of cells, measured by cell counting, met the experimental requirements, the next step of the research could be carried out. The PTCs used in this experiment were between 3 and 12 generations. At 24 h after cell seeding, the PTCs were treated with 5 μmol/L of the DNA methyltransferase (*DNMT1*) inhibitor 5- AzaCdR for 24 h.

### 2.2. Real-Time Quantitative PCR

Total RNA was isolated from PTCs using Trizol reagent (CWBIO, Beijing, China) according to the manufacturer’s instructions and was stored at −80 °C until use. About 1 μg of total RNA was used to synthesize first-strand cDNA using the PrimeScript™ RT Reagent Kit with gDNA Eraser (Takara, Beijing, China) following the manufacturer’s instructions. An equal volume of cDNA was used for qRT-PCR. qRT-PCR was performed in triplicate using a SYBR^®^ Premix Ex TaqTM II Kit (Takara, Beijing, China) on an ABI 7500 Real-Time PCR system. Gene expression levels were normalized to those of GAPDH, and fold change was determined using the 2^−∆∆Ct^ method [12]. In the qRT-PCR program, pre-denaturation at 95 °C for 5 min, denaturation at 95 ° C for 30 s, annealing at 60 °C for 30 s, extension at 72 °C for 30 s, and the number of cycles is 40.

### 2.3. WB Analysis

Total protein was extracted, and its concentration was detected using a BCA Protein Assay Kit (Vigorous Bio-technology Beijing Co., Ltd., Beijing, China). Equal amounts of sample protein were electrophoresed on 4–20% sodium dodecyl sulfate-polyacrylamide gels and transferred to polyvinylidene difluoride (PVDF) membranes. The PVDF membranes were then sealed with Tris-buffered saline-Tween (TBST) solution, which contained 5% skimmed milk, at room temperature for 1–2 h. After the PVDF membranes were washed with TBST three times, the membranes were incubated overnight at 4 °C with diluted primary antibodies for GAPDH (Servicebio, GB15004), DNMT1 (Affinity, DF7376), and SOD2 (Servicebio, GB111875), followed by incubation with secondary antibody HRP-conjugated Goat Anti-Rabbit IgG (Servicebio, GB23303).

### 2.4. SOD2 Gene Bioinformatics Analysis

The core promoter region of *SOD2* gene was predicted using Promoter 2.0 Prediction Server (http://www.cbs.dtu.dk/services/Promoter/) (accessed on 3 October 2024) and the online software from Berkeley Drosophila Genome Project (BDGP) (http://www.fruitfly.org/seq_tools/promoter.html) (accessed on 4 October 2024), FPROM and TSSW (http://www.softberry.com/) (accessed on 4 October 2024). The possible CpG islands were predicted using the program CpG Island Prediction within MethPrimer (http://www.urogene.org/methprimer/index1.html) (accessed on 16 August 2024). Use the online transcription factor prediction site AliBaba (http://www.gene-regulation.com/pub/programs/alibaba2/index.html) (accessed on 21 October 2024), and JASPAR 2018 (http://jaspar.genereg.net) (accessed on 22 October 2024) to predict transcription factors combining with the *SOD2* core promoter, with the threshold value set to 90%.

### 2.5. Construction of the Promoter Reporter

Total genomic DNA was extracted from PTCs according to the manufacturer’s protocol using the TIANamp Genomic DNA Kit (TIANGEN, Beijing, China) and was stored at −20 °C. The predicted promoter regions of SOD2 was cloned into pGL3-Basic recombinant vectors using seamless cloning. Specific primer information for the pGL3-Basic recombinant vectors, including sequences for the restriction enzyme sites Kpn I and Xho I and part of the vector sequences, is shown in Table 1 and Table 2. First, the target promoter fragment was amplified by PCR. Nine different truncated segments of the SOD2 promoter was amplified using PrimeSTAR^®^ Max DNA Polymerase (Takara, Beijing, China), with restriction enzyme sites and part of the vector sequences introduced by specific primers. Second, vector linearization was performed using restriction enzymes. The pGL3-Basic vectors were digested with restriction fast-cut enzymes Kpn I and Xho I (Takara, Beijing, China). Third, a seamless cloning reaction was performed. We made recombination between linearized vector and the truncated segments with a seamless cloning kit (Sangon, Shanghai, China). The ligated products were used to infect competent cells DH5α, and then spread on ampicillin selective medium overnight at 37 °C. The positive monoclonal colonies were picked and cultured at 37 °C with shaking at 200 r min^−1^ for 20 h. The culture solutions were examined by PCR, and then used to extract plasmids. The recombinant plasmids were constructed and detected by double enzyme digestion, respectively. Positive clones of recombinant plasmids were examined by sequencing (Shanghai Bioengineering Co., Ltd., Shanghai, China). We confirmed all constructs by Sanger sequencing. The pGL3-Basic recombinant vectors were named pGL3-Basic, P1, P2, P3, P4. (Table 3).

### 2.6. Detection of Dual Luciferase Activity

According to Lipofectamine 2000 Transfection System Instructions, PTCs cell line was cultured in DMEM/F12 containing 10% fetal bovine serum. The well-grown PTCs cell line was inoculated in a 24-well plate with three complex wells at a density of 1 × 10^5^ cells per well under 37 °C and 5% CO_2_. When monolayer cells reached to 70–80% confluence, plasmid transfection was performed. The Dual Luciferase Reporter Assay Kit (Vazyme, Beijing, China) was conducted following the manufacturer’s instructions. The ratio of Firefly-Luc/Renilla Luc was calculated to analyze the promoter activity using the ratio of empty vector PGL3-basic as a negative control.

### 2.7. Bisulfite Sequencing Polymerase Chain Reaction

Promoters were categorized into CpG island (CGI) and non-CpG island (NCGI) regions based on the distribution of CpG islands. Primer sequences were designed using MethPrimer, and the PCR products were analyzed by Sanger sequencing. Genomic DNA was extracted using the TIANamp Genomic DNA Kit (#DP304, TIANGEN BIOTECH, Beijing, China), and bisulfite conversion was performed with the DNA Bisulfite Conversion Kit (#DP215, TIANGEN BIOTECH, Beijing, China). The bisulfite-treated DNA was subsequently eluted with nuclease-free water. The *SOD2* promoter was amplified by polymerase chain reaction (PCR) using the methylation-specific PCR Kit (EM101, TIANGEN).

### 2.8. Promoter Fragment Methylation and Activity Measurement

The PCR product, designed with primers containing enzyme cleavage sites using NCBI, was cloned into the PGL-3 vector, with pGL3-basic and pGL3-control used as the control group. Then, the CpG methyltransferase M.SssI (EM0821, Thermo Fisher, Beijing, China) was incubated with these 9 vectors for 1 h at 37 °C to methylate all CpG residues, and the corresponding unmethylated group was incubated with nuclease-free water. The Hha I endonuclease was incubated with these vectors for 1 h at 60 °C to detect the methylation status. Luciferase activities were measured using a dual luciferase reporter gene assay kit and normalized to Renilla luciferase activity.

### 2.9. Statistical Analysis

One-way analysis of variance and the least significant difference method were used for variance analysis and multiple comparison of SOD2 expression using SPSS 25 (Version 25.0, IBM Corporation, Armonk, NY, USA). Each replicate was considered an experimental unit. Pearson’s correlation was used to analyze the correlation between the methylation level of CpG sites and gene expression.

## 3. Results

### 3.1. Identification of the Active Area of the SOD2 Promoter Region

**Figure 1:**
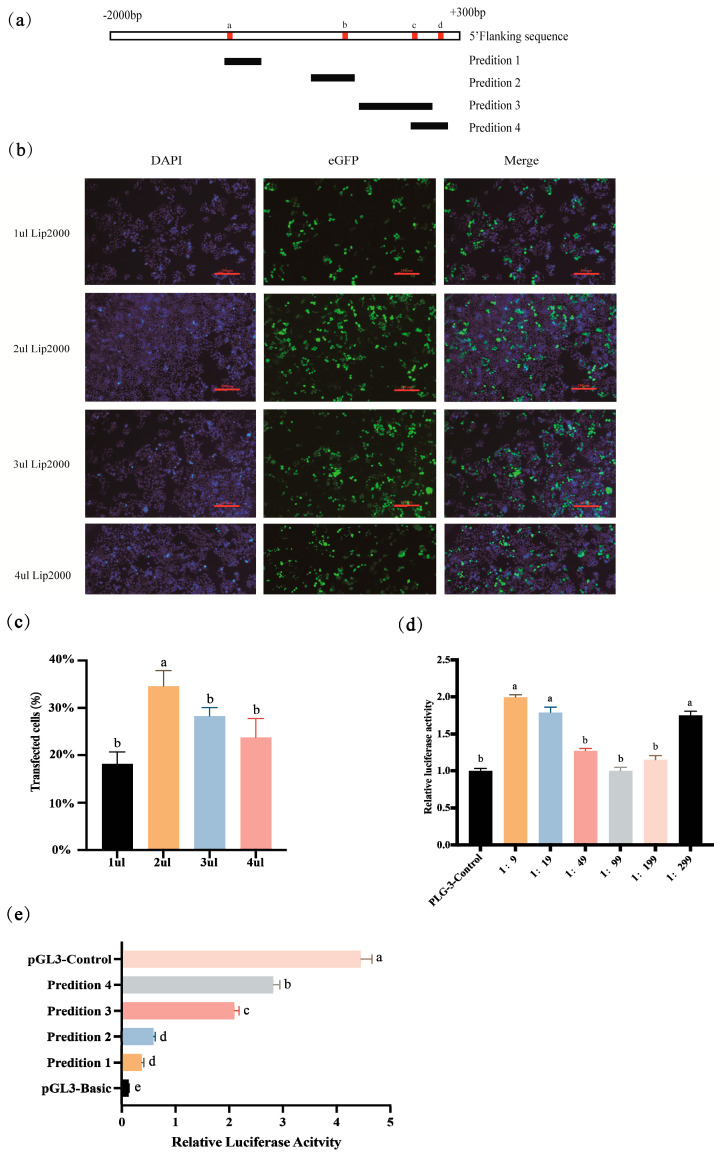
Determination of the core promoter of porcine *SOD2* gene. (**a**) Design of core promoter prediction fragments of *SOD2* gene. The transcription start site is 0 bp. The red dots represent the core promoter. a corresponds to the −1300 bp position predicted in Table 4, b corresponds to −629–579 bp, c corresponds to 178–228 bp and 212 bp, and d corresponds to 100 bp. (**b**) Cells were transfected with green fluorescent plasmids (eGFP) and Lip2000 liposomes of different concentrations. Scale bar, 200 μm (**c**) Statistics of green fluorescent cells measured by Image J. (**d**) The transfection efficiency of pGL3-Control and pRL-TK at different ratios. (**e**) Double luciferase activity of *SOD2* gene promoter fragment. Different letters (a–e) indicate significant differences at *p* < 0.05 (n = 3).

To identify the core promoter regions regulating SOD2 transcription, primers was designed to construct a vector containing the promoter regions based on software-predicted locations (Figure 1a, Table 4). The promoter fragment was ligated to the linearized pGL3-basic vector, and PTCs were co-transfected with the recombinant plasmid and the pRL-TK vector. To select the optimal dose of Lipofectamine 2000 for lipid transfection, concentrations ranging from 1 µL to 4 µL were tested in PTCs transfected with the eGFP plasmid. A dosage of 2 µL produced the highest GFP fluorescence (Figure 1b,c). To screen for the optimal ratio of the target plasmid and the internal reference plasmid pRL-TK, the plasmid to liposome ratio of 500 ng: 2 μL was continued to be used to detect the firefly luciferase values at different ratios of pGL3-Control and pRL-TK plasmids. Six different ratios of pGL3-Control to TK were tested. The results showed that a 1:99 pGL3-Control to TK ratio had the least impact on the system, with activity levels comparable to those of the control group (Figure 1d).

P1 contains core promoter a, P2 contains core promoter b, P3 contains core promoter c, and P4 contains core promoters c and d. The results showed that the *SOD2* promoter fragments displayed varying activities in PTCs. For the *SOD2* promoter, P4 (*SOD2* + 76/+244) showed the highest activity (Figure 1e).

### 3.2. Binding of Transcription Factor SP1 to the Core Promoters of SOD2

**Figure 2:**
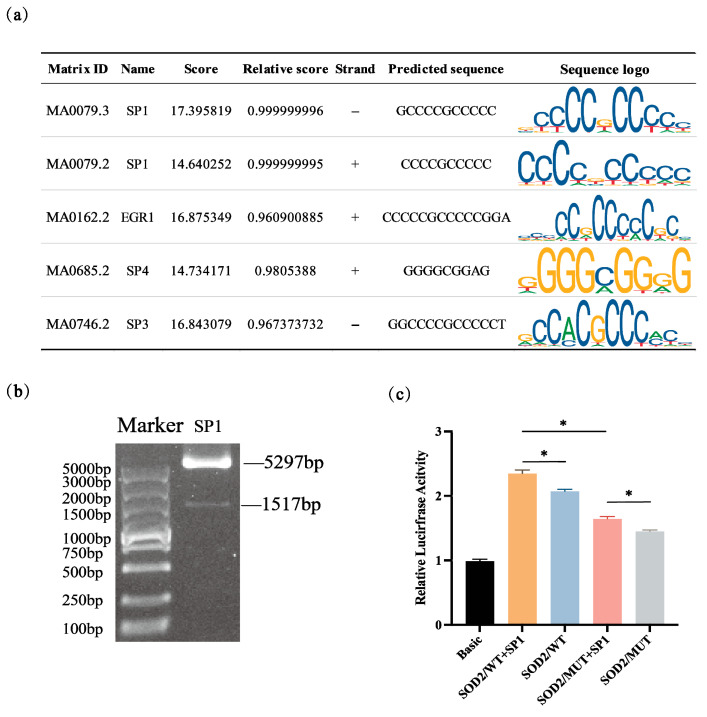
SP1 Binds to the Core Promoter of SOD2. (**a**) Prediction of transcription factors in the core promoters of *SOD2* gene. (**b**) Construction of SP1 overexpression vector. (**c**) Co-transfection of transcription factor SP1 with wild-type and mutant *SOD2* core promoter fragments dual luciferase activity. * indicates a significant difference at *p* < 0.05 (n = 3).

Based on the predicted core promoter sequence of the porcine SOD2 gene in P4, we predicted the potential transcription factor binding site (TFBS) using Animal TFDB (https://guolab.wchscu.cn/AnimalTFDB4/#/, accessed on 20 October 2025) and JASPAR (https://jaspar.genereg.net/). Results showed that, among the transcription factors binding to the core promoter of the *SOD2*, SP1 had two predicted binding sites with scores of 17.395819 and 14.640252 respectively (Figure 2a). To further confirm the involvement of binding site, the highest score predicted site has been selected and was mutated by site-specific mutagenesis (Table 1), and the corresponding expression vectors and SP1 expression vector were constructed (Figure 2b). PcDNA3.1-Sp1 and the core promoter vector were co-transfected into cells, and dual luciferase activity was measured. As expected, SP1 significantly enhanced the fluorescence activity of the plasmid containing the *SOD2* core promoter fragments (*p* < 0.05), whereas the mutant plasmid exhibited reduced fluorescence activity (Figure 2c).

### 3.3. 5-Aza-CdR Treating Promotes the Expression of SOD2 in PTCs

**Figure 3:**
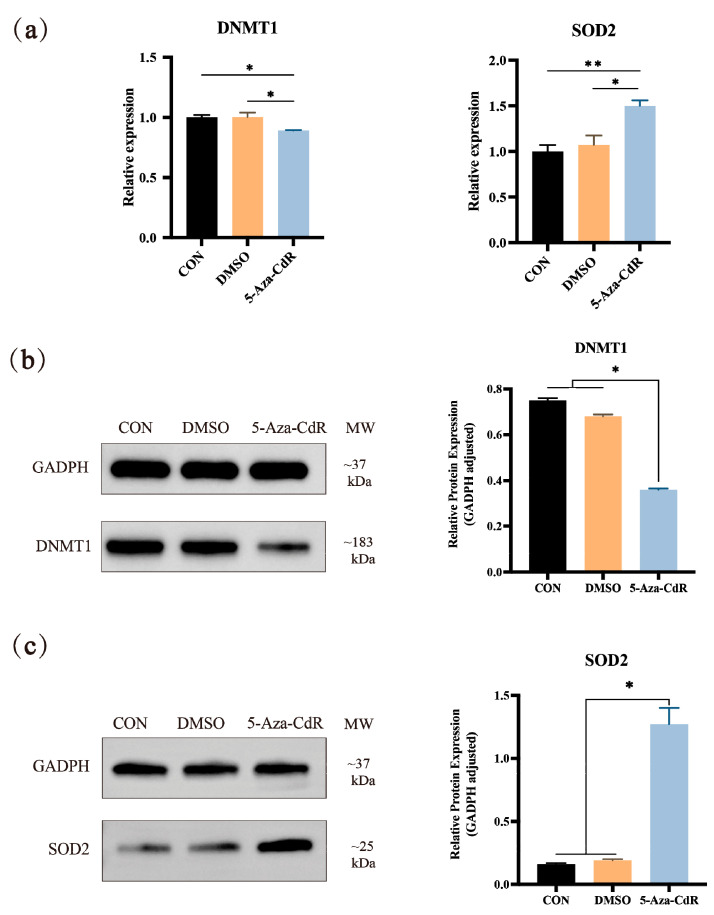
5-Aza-CdR Treatment Induces SOD2 Expression in PTCs. (**a**) The mRNA expression level of *SOD2* and *DNMT1* in PTCs treated with 5-Aza-CdR. The protein expression level of DNMT1 (**b**) and SOD2 (**c**) in PTCs treated with 5-Aza-CdR. The band intensities were measured by Image J (v1.53) and normalized to GADPH. * indicates a significant difference at *p* < 0.05, ** indicates a significant difference at *p* < 0.01 (n = 3).

To confirm the regulation of DNA methylation on the expression of *SOD2*, the expression of *SOD2*, *DNMT1*, in PTCs were examined after treatment with DNA methylation inhibitor (5-Aza-CDR). qRT-PCR showed that *DNMT1* gene expression was significantly decreased (*p* < 0.05), at the same time *SOD2* gene expression was significantly increased (*p* < 0.05) (Figure 3a). Western blot analysis demonstrated a significant increase in *SOD2* protein expression levels (*p* < 0.05) (Figure 3b).

### 3.4. Promoter Hypomethylation Promotes SOD2 Expression

**Figure 4:**
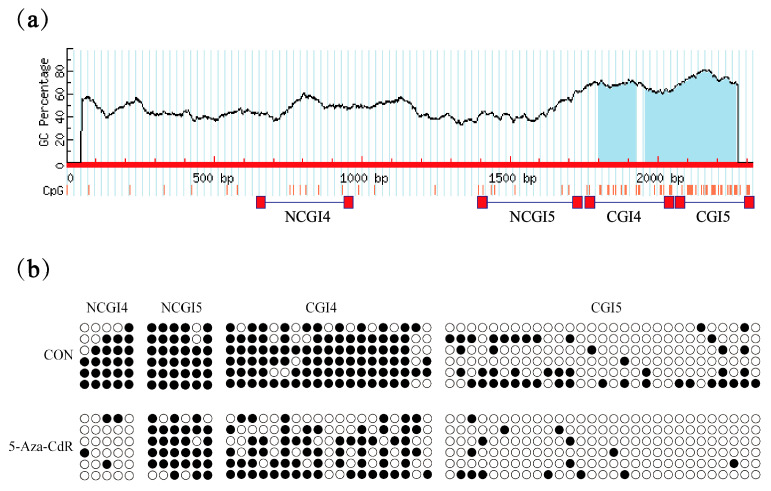
SOD2 Upregulation is Driven by Promoter Hypomethylation (**a**) Schematic distribution of the CpG islands and bisulfite sequencing primers in the promoter of *SOD2*. Blue region is the CpG island; red and horizontal bars show the positions of bisulfite primers. (**b**) CGI CpG island, NCGI non-CpG island. BSP was used to detect the NCGI and CGI methylation status of PTCs in porcine treated with 5-Aza-CdR.

To investigate the role of DNA methylation in the expression of *SOD2*, the methylation status of the promoters was assessed. The promoter regions were divided into CpG island (CGI) and Non-GpG island (NCGI) regions based on the site-predicted promoter cpg island positions, (http://www.urogene.org/methprimer/index1.html, accessed on 20 October 2025) (Figure 4a) we constructed the corresponding expression vectors (Table 5 and Table 6). Bisulfite sequencing PCR (BSP) was employed to assess the methylation status of the CGI and NCGI regions. The results showed that PTCs treated with 5-Aza-CdR significantly reduced the methylation levels of CGIs and NCGIs at *SOD2* promoters (Figure 4b).

### 3.5. Hypermethylation Reduces Promoter CGI5 Activity

**Figure 5:**
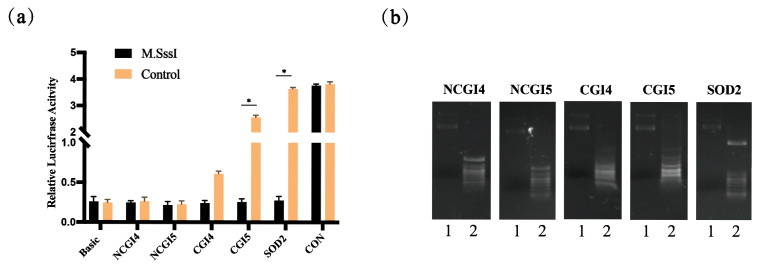
Hypermethylation Represses CGI5 Promoter Activity(**a**) The luciferase reporter plasmids carrying different regions of *SOD2* were methylated in vitro and transfected into PTCs. Relative luciferase activity in PTCs were determined. * indicates a significant difference at *p* < 0.05. (**b**) Gel electrophoresis of fragments treated with CpG methyltransferase M.SssI and digested with Hha I, 1 represents the treated with M.SssI, and 2 represents the untreated with M.SssI. * indicates a significant difference at *p* < 0.05 (n = 3).

The effect of DNA methylation on the *SOD2* promoter activity was investigated. We used CpG methyltransferase M.SssI to treat the constructed CGI and NCGI vectors to induce methylation. The effect of methylation was verified by treating the vector with Hha I endonuclease. The methylated CG sites could not be cleaved by Hha I endonuclease. The gel electrophoresis bands of the M.SSI treatment group were intact, while those of the control group were cleaved into small sequences by Hha I endonuclease, indicating the hypermethylation of the vector. Gel electrophoresis results showed distinct bands, confirming the successful methylation effect (Figure 5b). Dual luciferase activity analysis showed that the treatment group significantly reduced the transcriptional activity of the CGI5 fragment of *SOD2* compared to the control group. However, methylation had no significant effect on the transcriptional activity of the NCGI promoter region (Figure 5a).

## 4. Discussion

Oxidative stress significantly impacts the reproductive performance of livestock, particularly by impairing placental function. The placenta, serving as a critical link between the mother and fetus, is susceptible to oxidative stress. This leads to decreased nutrient delivery efficiency, structural alterations, and abnormal fetal development [13]. SOD is a key antioxidant enzyme in pigs and other livestock, essential for controlling oxidative stress and maintaining physiological functions [14]. SOD is extensively studied for its application in reducing oxidative damage and as a biomarker in oxidative stress-related diseases. It is critical for cellular defense mechanisms and holds therapeutic potential [15]. DNA methylation refers to the binding of a methyl group to the DNA, mainly at positions where a cytosine is located next to a guanine, a cytosine phosphate-guanine (CpG) site [16]. DNA methylation plays a crucial regulatory role in the expression of SOD genes. The methylation status of genes is both a key mechanism for regulating gene expression and a potential target for the treatment of oxidative stress-related diseases [17]. Promoter hypermethylation typically inhibits gene expression, while hypomethylation can activate SOD gene expression. In the placenta of patients with preeclampsia, *SOD1* gene was found to be abnormally methylated, and its expression was significantly decreased [18]. Furthermore, studies have shown that the reduction in DNMT-mediated CpG island methylation level in human pulmonary artery smooth muscle cells can increase the expression of *SOD2* [19]. Although there are numerous studies reporting on the expression regulation of *SOD2* in various species, limited research exists on the regulation of SOD methylation expression in pigs.

The core promoter is a region near the transcription start site, containing key regulatory elements like the TATA box and Inr. These elements are responsible for recruiting transcription factors and RNA polymerase II to initiate the gene transcription process [20,21]. In this study, we identified the core promoters of *SOD2* gene and analyzed the methylation status of these promoters. First, we determined the core promoter regions of those genes, which can recruit transcription factors to initiate transcription. Multiple bioinformatics tools were used to predict the core promoter of *SOD2* gene. We selected several high-scoring results and constructed truncated promoter segments and identified the core promoter region through the Dual-Luciferase^®^ reporter assay system for Renilla and firefly luciferase activity. We found that the P4(*SOD2* + 76/+244) regions of the *SOD2* was significantly higher than that in other regions. Therefore, we determined that the two regions were the core active areas of the promoter.

Transcription factors within the core promoter modulate gene transcriptional activity by regulating RNA polymerase recruitment and transcription initiation [22]. Transcription factors within the core promoters were further predicted, and the results showed that SP1 had the highest score for both genes. SP1 is a well-known transcription factor involved in regulating gene transcription. Studies have shown that SP1 directly regulates the transcription of several antioxidant genes, contributing to antioxidant activity [23]. We then designed and constructed vectors for the two core promoters with SP1 transcription factor binding site mutations and expression vector of SP1. As expected, after co-transfection with SP1, the dual luciferase activity of the mutant core promoter was significantly lower than that of the wild-type, but it was significantly higher than in cells transfected with the core promoter fragment alone. These results confirmed that SP1 binds to both core promoters and influences the transcription process.

Porcine trophoblast cells are a crucial cell type in the pig placenta, responsible for forming the fetal compartment. These cells play a key role in nutrient exchange between the fetus and the mother, immune regulation, and maintaining placental structure [24]. To investigate the regulatory effect of DNA methylation on placental antioxidants and its impact on *SOD2* gene expression, PTCs were chosen to explore the underlying mechanisms. The primary DNA methyltransferases identified in mammalian cells include *DNMT1*, *DNMT2*, and the *DNMT3* family [25]. *DNMT1*, in particular, is responsible for initiating de novo methylation [26]. Therefore, we treated the cells with 5-Aza-CdR to reduce *DNMT1* levels, and subsequently measured the expression of *SOD2*, and *DNMT1* to investigate the effect of DNA methylation on placental antioxidants. The results showed that, compared with the control group, the expression of the SOD2 gene increased significantly and the expression of DNMT1 decreased significantly. We will next analyze the mechanism through which methylation influences gene expression.

Recent studies have revealed that promoter methylation is a crucial mechanism for regulating gene expression [27] and plays a significant role in the development of diseases such as cancer and neurodegenerative disorders [28]. When CpG islands in the promoter region are methylated, the binding of transcription factors and RNA polymerase is hindered, leading to reduced transcriptional activity. In contrast, demethylation can relieve this repression and promote gene expression [29]. For example, studies have found that the hypermethylation of the *SOD1* gene promoter region significantly reduces its expression in oxidative stress, which is closely related to the occurrence of disease [30,31], and aberrant promoter methylation of antioxidant genes, including *SOD1*, can disrupt the cellular redox balance and contribute to various pathological conditions [32,33]. We used the BSP method to assess the methylation status of the *SOD2* promoter in cells treated with 5-Aza-CdR. The *SOD2* promoters were fragmented, and the corresponding vectors were constructed. The results indicated that the promoter methylation levels in the 5-Aza-CdR treatment group were significantly lower than those in the control group. This corresponds to an increase in *SOD2* expression levels

DNA methylation in the promoter region plays a crucial role in regulating gene expression, with hypermethylation often linked to the suppression of promoter activity [34]. Hypermethylation of the promoter region inhibits the transcriptional activity of the gene by interfering with the binding of transcription factors and reducing the interaction between transcription factors and promoters [35]. The methylation status of genes is not only an important mechanism for regulating gene expression but also a potential target for the treatment of oxidative stress-related diseases [36,37]. In this experiment, CGI and NCGI fragments were treated with CpG methyltransferase M.SssI and transfected into cells for dual luciferase activity detection. Significant differences were observed CGI5 of *SOD2* gene between the treatment and control groups. In addition, the fragment CGI5 with significant differences in activity were almost consistent with the core promoters P4 identified in the first part. These results offer insight into how epigenetic modifications influence the transcriptional activity of pig *SOD2* and its role in oxidative stress regulation, further supporting the idea that promoter methylation is a key mechanism regulating gene expression.

## 5. Conclusions

The results of this study indicated that the P4(*SOD2* + 76/+244) region of the *SOD2* promoter region has strong promoter activity. Furthermore, the expression of SOD2 is regulated by methylation of the core promoters in the CpG islands. Hypomethylation promotes SOD expression, while hypermethylation decreases promoter activity. In addition, transcription factor binding sites have been identified in the SOD2 promoters, with the SP1 binding site being the most prominent, potentially playing a significant role.

## Figures and Tables

**Table 1:** The sequence of pGL3-SOD2.

Supporter Name	Sequence
pGL3- SOD 2 (Wt)	CTCGAGCCAACTTCGTGTGCTGCAATCCAGGATTGCCCGTTTTTGAGGAGAAATGTTGTTTTTTTCCACAGCGCCAGCTTTCTGGGCGTTTTACAGCCGCGGGGCGGTGGGAGCCGGCGGCGTGGAGAGGAAGGCTGCACACGCGGCCCCTTGGCCCCGCCCCCGGCCATTCCCGGCCGCTCGCGTCCCGAGGTTCCCCGTGGGGGCGGGTCCATGG CTCGAGCCAACTTCGTGTGTGCAATCCAGGATTGCCCGTTTTGA
pGL3- SOD 2 (Mut)	CTCGAGCCAACTTCGTGTGCTGCAATCCACGGATTGCCCGTTTTTGAGGAGAAATGTTGTTTTTTTCCACAGCGCCAGCTTTCTGGGCGTTTTACAGCCGCGGGGCGGTGGGAGCCGGCGGCGTGGAGAGGAAGGCTGCACACGCGGCCCCTTGCGGGGAGGGGCGGCCATTCCCGGCCGCTCGCGTCCCGAGG

* The underlined part is the restriction site sequence, and the blue part is the predicted site sequence binding to the transcription factor. The gray part represents the mutant sequence of the predicted transcription factor binding site.

**Table 2:** The sequence and primer name.

Primers Name	Primer Sequence 5′-3′	Product Length (bp)
pGL3-sod2F	AACGGGCCCTCTAGACTCGAGCGCGGCAGCTGGAAAAAG	169
pGL3-sod2R	TTTAAACTTAAGCTTGGTACCTGGCTCCGCCCCCTACGC	169
PcDNA3.1-SP1F	AACGGGCCCTCTAGACTCGAGGAGTCCCAGCCATCCCCTT	1517
PcDNA3.1-SP1R	TTTAAACTTAAGCTTGGTACCCTGAGGCATTTGCTATAGCCAA	1517

* The shadow part is the seamless clone overlap sequence, and the underlined part is the restriction site sequence.

**Table 3:** Primers for constructing core promoter vector.

Primers	Primer Sequence 5′-3′	Product Length (bp)
P1 (*SOD2*)	**F**:CagaacatttctctatcgataggtaccGGCACTGGCCCTACATCTTAG **R:**AagcttacttagatcgcagatctcgagATGGTATGGCCCTAAGCCTCC	217
P2 (*SOD2*)	**F:**CagaacatttctctatcgataggtaccAACCGGGACGCTAGTGCAG **R:**AagcttacttagatcgcagatctcgagCCCGAGACGCGCTACAAG	233
P3 (*SOD2*)	**F:**CagaacatttctctatcgataggtaccGTTCAGCGAAGACCCAACTC **R**:AagcttacttagatcgcagatctcgagGCCTTAGCAGCGAGGTCTTG	398
P4 (*SOD2*)	**F:**CagaacatttctctatcgataggtaccCAGTTCTTGCGTCCCCTGAG **R**:AagcttacttagatcgcagatctcgagGAGACGCGCTACAAGGACAT	169

* The underlined part is the restriction site sequence.

**Table 4:** Prediction of core promoter of *SOD2* gene.

Prediction Software	Promoter Position (bp)	Score
BDGP (v2.2)	−629–579 (b)	0.87
	178–228 (c)	0.86
Promoter 2.0	−1300 (a)	0.6
	100 (d)	0.579
TSSW	212 (c)	8.85

* The abcd in the table corresponds to that in Figure 1a.

**Table 5:** Primers used for vector construction.

Primers	Primer Sequence 5′-3′
BSP-*SOD2* NCGI4	**F**: ATGCTCCTCAGTTTCCCATTACT **R**: AGGTGGGGTTGGCTGATAGT
BSP-*SOD2* NCGI5	**F**: CCTCCGTTGTACTTTTATATTCTCC **R**: C TCGCAGTCCCCCCCTCATAT
BSP-*SOD2* CGI4	**F**: ACTGGCCCTACATCTTAGCA **R**: TCGCAGTCCCCCCCTCATAT
BSP-*SOD2* CGI5	**F**:TTGGAAAAAGTGTAGGTGTTTTAGTTT **R**:TAAACCCTCAAAATTCCAACC

**Table 6:** Primers for constructing core promoter vector.

Primers	Primer Sequence 5′-3′	Product Length (bp)
NCGI4	**F**:ATTTCTCTATCGATAGGTACCTACTAAGCACATGTTTGAGATTTATTTATTAT **R**:ACTTAGATCGCAGATCTCGAGTCACAGCAAGAAGGTGGGGT	365
NCGI5	**F**:CAGAACATTTCTCTATCGATAGGTACCGTTCAGCGAAGACCCAACTC **R**:AAGCTTACTTAGATCGCAGATCTCGAGGCCTTAGCAGCGAGGTCTTG	218
CGI4	**F**:ACTTAGATCGCAGATCTCGAGGTGACCTTGCTTTCCGAGTAAAC **R**:ATTTCTCTATCGATAGGTACCAGACTCCTCCGTTGTACTTTTATATTCT	310
CGI5	**F**:CAGAACATTTCTCTATCGATAGGTACCCAGTTCTTGCGTCCCCTGAG **R**:AAGCTTACTTAGATCGCAGATCTCGAGGAGACGCGCTACAAGGACAT	169
*SOD2*	**F**:ATTTCTCTATCGATAGGTACCTATGGAAGGGAAATTCCTCATAGTG **R**:ACTTAGATCGCAGATCTCGAGTGGACCCTCAGAATTCCAGCC	2300

* The underlined part is the restriction site sequence.

## Data Availability

The original contributions presented in this study are included in the article/Supplementary Materials. Further inquiries can be directed to the corresponding author.

## Supplementary Materials

The following supporting information can be downloaded at: https://www.mdpi.com/article/10.3390/vetsci12121133/s1, Figure S1: original image of Figure 2b; Figure S2: original image of Figure 3b; Figure S3: original image of Figure 3c; Figure S4: original image of Figure 5b.
