# Supplementary material for: Core Promoters of Pig SOD2 Gene and Its Expression Regulation by DNA Methylation
Source: Vet Sci. 2025 Nov 28;12(12):1133. doi: 10.3390/vetsci12121133 (PMC12737695; doi:10.3390/vetsci12121133)

Core Promoters of Pig SOD2 Gene and Its Expression Regulation by DNA Methylation

(photo original image Western Blot figure 2b)

Figure S1: original image of figure 2b

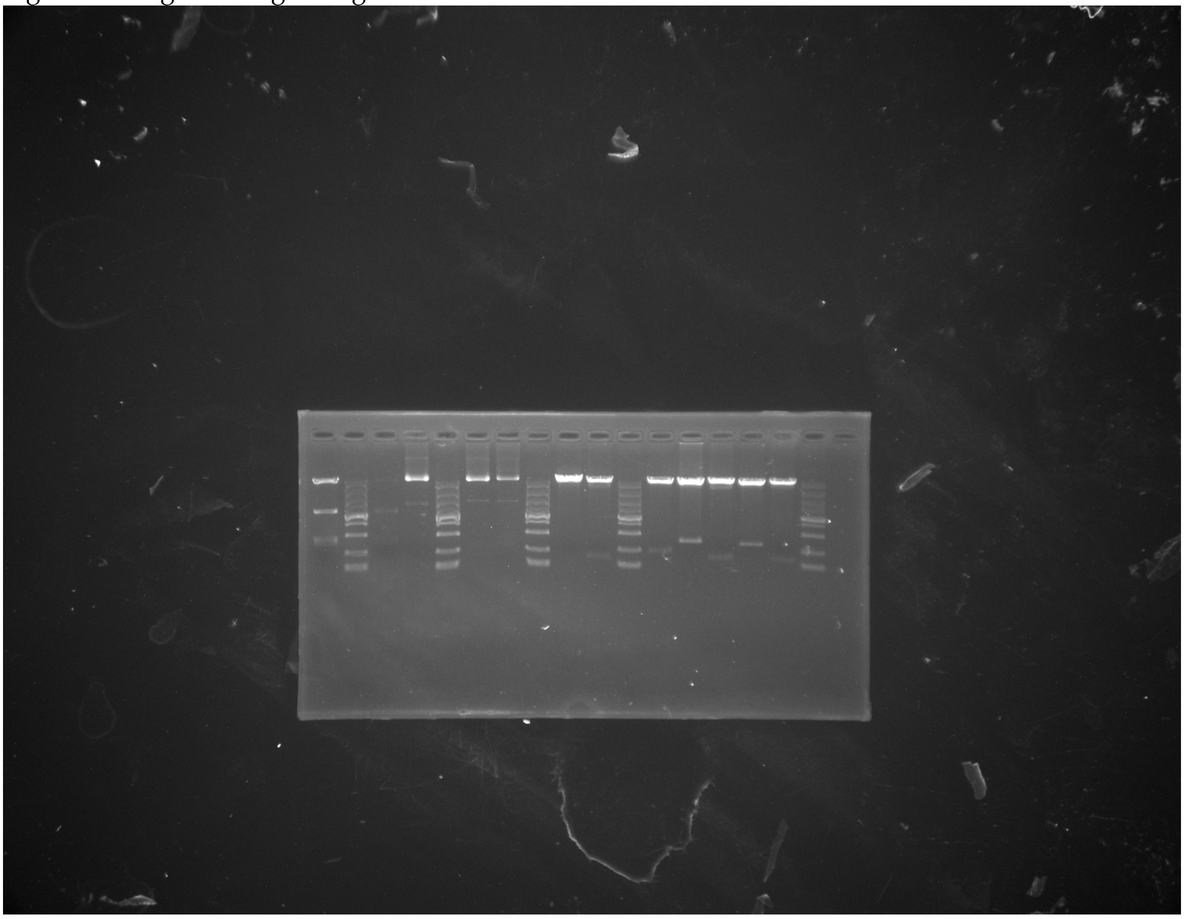

(photo original image Western Blot figure 3b)

Figure S2: original image of figure 3b

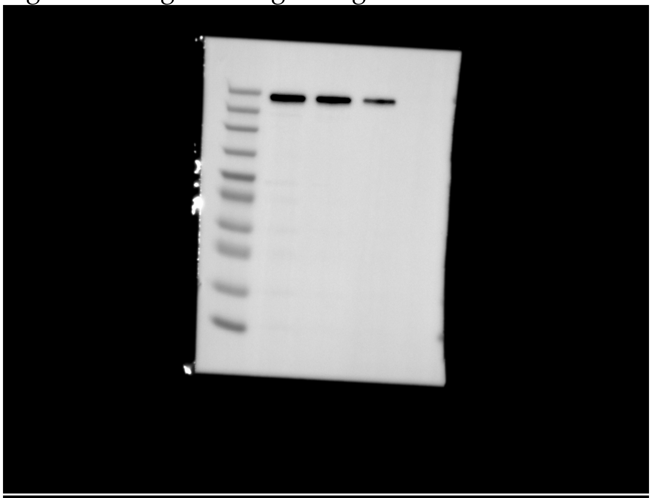

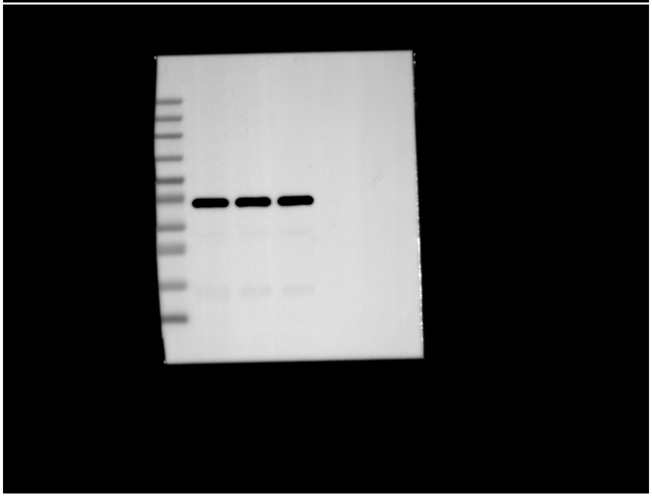

(photo original image Western Blot figure 3c)

Figure S3: original image of figure 3c

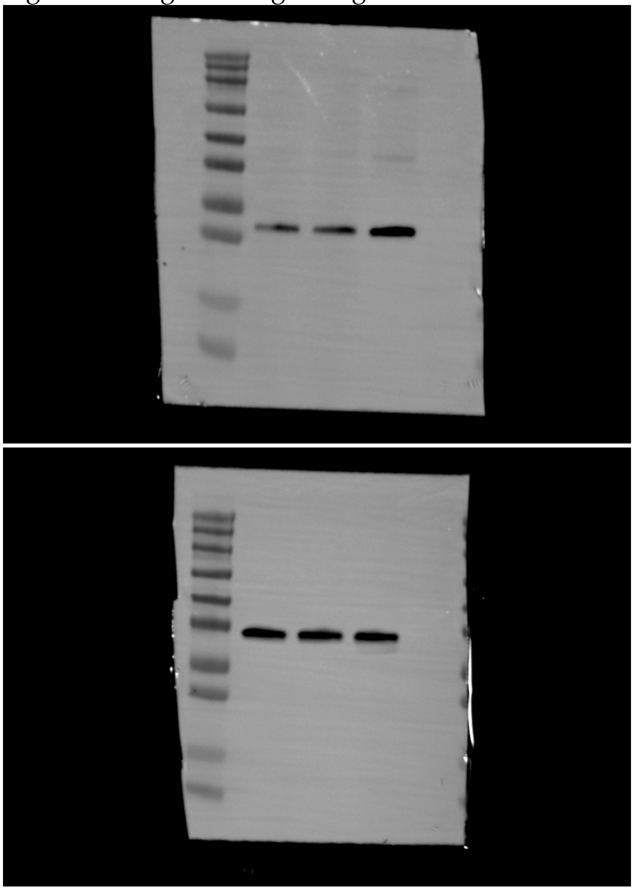

(photo original image Western Blot figure 5b)

Figure S4: original image of figure 5b

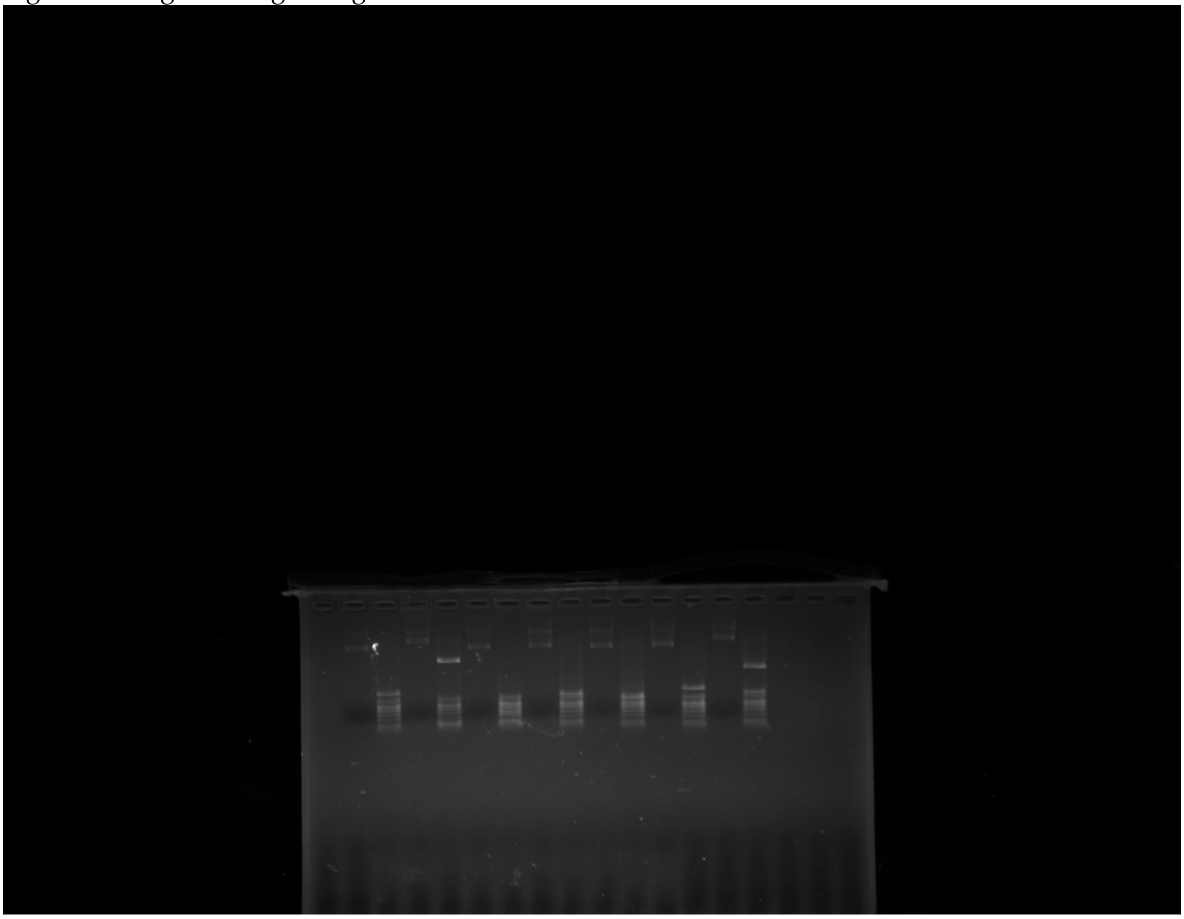

Supplement: Supplementary file 1 [file vetsci-12-01133-s001.zip › vetsci-3969741-supplementary.pdf]
